# Materials in Cultural Heritage: Analysis, Testing, and Preservation

**DOI:** 10.3390/ma18194540

**Published:** 2025-09-29

**Authors:** Žiga Šmit, Eva Menart

**Affiliations:** 1 Faculty of Mathematics and Physics, University of Ljubljana, Jadranska 19, SI-1000 Ljubljana, Slovenia; 2 Department of Low and Medium Energy Physics, Jožef Stefan Institute, Jamova 39, SI-1000 Ljubljana, Slovenia; eva.menart@nms.si; 3 National Museum of Slovenia, Prešernova 20, SI-1000 Ljubljana, Slovenia

Many things that people achieved in the past are worth admiration, so we are prone to keep, nurse, and preserve them. We can also learn from examples in the past, though many experiences deliberately remain forgotten. The development of analytical techniques has enabled further possibilities for studying how people created, produced, and manipulated certain objects, which then facilitated studies in social sciences and humanities including history, cultural anthropology, or art history. Analytical studies then aim in two directions: the first is to discover properties of the objects that enable their more precise characterization according to their composition, age, or other properties, while the second direction aims toward the preservation of objects, so the analysis tries to uncover the formation of harmful compounds or the degree of degradation. This type of division is also reflected in our volume, so we will first introduce the papers that deal with analytical characterization, followed by papers concerned with conservation; we also distinguish by the historical period.

Clay was the first material than man chemically modified with thermal treatment. The Longshan Culture in Eastern Henan was already studied in the 1990s [1], but knowledge was further expanded in a study by Linyu Xia et al., where the authors employed XRF, XRD, infrared spectroscopy, and SEM-EDS to study Neolithic pottery of the Longshan period from three sites in the Yongcheng area, pointing to the specific differences and cultural relations among them. Slightly younger is the Eneolithic pottery from Velika Humska Čuka near Niš in present-day Serbia [2], which was also investigated by the authors Maja Gajić-Kvaščev et al., who employed EDXRF in combination with statistical techniques, which are indispensable tools in many heritage science studies [3,4]. The aim was to distinguish ceramics using local clay from deposits near the site from imported items, testifying to a long-range connection and exchanges. In the ongoing work, the same authors examined provenance studies of prehistoric ceramics using portable XRF and advanced statistical techniques. Besides artifacts, bone remains can reveal details of people’s life such as diet, possible migrations, and even the type of burial (e.g., cremation) [5]. Tamara Leskovar et al. studied the changes in bone exposed to high temperature (caused, for example, during cremation of the deceased) using ATR-FTIR and computed tomography.

Glass and glassy materials are further artificial materials that have accompanied man since prehistory; during the Roman period, glass was a widely used consumable good, subject to a wide-range- trade [6]. Žiga Šmit and Tina Milavec studied the glass from Koper, a port at the northeastern Adriatic, pointing to the transition in the glass industry from the Late Antiquity until the Middle Ages, a theme that has been intensively studied in the Mediterranean. By adding small amounts of chromophores, glass can attain different colors. Amber glass has been known since prehistory, and was intensively used for the storage of food and medicaments until the end of the 19th century [7]. Catarin Reis Santos et al. tried to reproduce amber glass following historic recipes from the late 18th century. Enamels are glassy materials characterized by a lower melting point and are usually applied as thin layers on a metal or glassy substrate, which could be studied according to their refractive index [8], and was attempted by Teresa Palomar et al. The authors reviewed different mathematical models for refractive index in lead glasses; they further discuss the influence of glass corrosion and the appearance of alteration layers, with possible application in glass preservation.

Buildings are evident remains of past human activity. Raw walls are usually covered with plasters, and also with frescos in monumental buildings. Such structures in the temple city of Bagan in Ancient Burma have already been investigated [9], and further exploration was carried out by Hye Ri Yang et al. in their study of the calcareous materials at the Phaya Thon Zu Temple in Myanmar. The analysis of mortars showed that they were produced by mixing clay and sandy soil, and the location of the sandy soil for the newly produced plasters was proposed. Corbii de Piatră is a monastery in Romania, renowned for being carved into a huge sandstone rock and decorated with frescos [10]. Marioara Abrudeanu et al. studied the plaster support of the frescos by microscopy and compositional analysis. Carbon dating performed by AMS confirmed two production phases dated to the 14th century.

Analytical studies are particularly important for studies of materials affected by degradation, especially in harsh conditions such as a marine environment [11]. Liang Zheng et al. studied a metal nameplate on the Vila D. Bosco, a modern building from the former Portuguese colony in Macau. The authors applied several analytical techniques, such as XRF, SEM-EDS, XRD, and ATR-FTIR, to analyze the basic material and corrosion products. The plate was made of steel with a high sulfur content, which induced corrosion. The study proposes a restoration plan and protection methodology for objects exposed in a subtropical marine environment. For protection against volatile organic compounds and other environmental factors such as high humidity, hybrid aerogels have previously been investigated [12], and are now being further developed by George Gorgolis et al. as a three-dimensional aerogel based on graphene and transition-metal dichalcogenides, which can absorb harmful substances. The last set of investigated objects is from the darkest period of human history: belongings of the victims from the Nazi concentration camp Auschwitz-Birkenau. As textiles are prone to microbial deterioration [13], the authors Anna Wawrzyk et al. proposed the disinfection of cotton with ethanol mist for their protection and preservation. Subsequent investigation with FTIR-ATR and XPS showed no significant changes in the fabric.
